# Single‐dose pharmacokinetics of telmisartan oral solution and effect of feeding in dogs

**DOI:** 10.1111/jvp.13104

**Published:** 2022-11-10

**Authors:** Allison G. Bechtel, Jennifer M. Reinhart, Zhong Li

**Affiliations:** ^1^ The Department of Veterinary Clinical Medicine, College of Veterinary Medicine University of Illinois Urbana‐Champaign Urbana Illinois USA; ^2^ The Metabolomics Lab, Roy J. Carver Biotechnology Center University of Illinois Urbana‐Champaign Urbana Illinois USA; ^3^ Present address: Duke Proteomics and Metabolomics Shared Resource Duke University Durham North Carolina USA

**Keywords:** angiotensin II receptor blocker, dog, hypertension, pharmacokinetics, proteinuria

## Abstract

Telmisartan is an angiotensin II receptor blocker that has great potential to improve the treatment of hypertension, proteinuria, and cardiovascular disease in dogs. A feline‐approved telmisartan oral solution (TOS) is available, but this formulation has not been evaluated in dogs. The aims of this study were to establish the pharmacokinetics of telmisartan administered as TOS and determine the effect of feeding on drug absorption in dogs. In a cross‐over design, seven healthy dogs received 1 mg/kg telmisartan orally as TOS with or without food and underwent serial measurement of plasma telmisartan concentrations over 24 h. Bioequivalence of TOS administered with vs. without food was assessed by the 90% confidence interval method for maximum concentration (C_max_), and the observed and extrapolated areas under the curve (AUC_0–t_ and AUC_0–∞_). The mean ratios of these parameters were 0.97 (CI 0.74–1.27), 0.92 (0.81–1.03), and 0.90 (0.82–1.00), respectively. Feeding methods were not bioequivalent based on C_max_ due to interindividual variation. These results suggest that TOS can be given to dogs with or without food but should be administered in the same way consistently. Additional pharmacokinetic and pharmacodynamic studies are warranted to confirm this recommendation and establish the therapeutic targets for telmisartan in dogs.

## 1 INTRODUCTION

Telmisartan is a relatively new angiotensin II receptor blocking drug (ARB) used for the treatment of systemic hypertension (Acierno et al., 2018), proteinuria (Bugbee et al., 2014), renal disease (Lourenço et al., 2020), and cardiovascular disease (Kawaguchi et al., 2019) in dogs, cats, and humans (Michel et al., 2016). Veterinary interest in telmisartan is growing given the mechanistic benefits of ARBs over the traditional angiotensin converting enzyme inhibitors (ACEi) when suppression of the renin‐angiotensin‐aldosterone system (RAAS) is clinically indicated. ARBs might be superior to ACEi because they selectively inhibit the maladaptive effects of the angiotensin type I receptor (AT_1_R) with high affinity, while still allowing for the protective counterregulatory effects of AT_2_R; this is in contrast to the ACEi, which indirectly inhibit both AT_1_R and AT_2_R. This unique mechanism of action reduces aldosterone synthesis to promote vasodilation, natriuresis and improved attenuation of the pressor response to angiotensin II (Ames et al., 2019; Jenkins et al., 2015), allowing ARBs (telmisartan) to be considered as the first line therapy for diseases requiring RAAS modulation in dogs (Brown et al., 2013).

Telmisartan oral tablets (Micardis®, Boehringer Ingelheim International GmbH) are currently the most commonly used telmisartan formulation in canine medicine. In 2018, telmisartan oral solution (TOS, Semintra®, Boehringer Ingelheim Vetmedica GmbH) gained FDA approval for the treatment of feline systemic hypertension (Food and Drug Administration, 2018). TOS is commercially available in the United States formulated as a non‐flavored, viscous, 10 mg/ml oral liquid solution. This low concentration allows more accurate dosing in small breed dogs. Additionally, the new liquid formulation may provide a more attractive option to owners of dogs that resist oral tablet administration, which could improve overall compliance. However, the pharmacokinetics of TOS in dogs are currently unknown, precluding rational guidelines for the use of this product in this species.

Pharmacokinetic profiles have been established for telmisartan oral tablets in dogs (Baek et al., 2013). However, the data cannot be extrapolated to TOS in dogs because differences in formulation could affect parameters, particularly those affected by absorption (Riviere, 2009). Similarly, the pharmacokinetics of TOS have been evaluated in cats, but these cannot be extrapolated to dogs owning to species‐specific differences in drug disposition (FDA, 2018; Sutton, 2004). The effect of feeding on absorption must also be critically evaluated in dogs with respect to the specific dose and formulation investigated before accurate dosing recommendations can be advised. In humans and cats, the oral bioavailability of telmisartan is reduced when given with food. This difference did not affect primary outcomes in phase 3 clinical trials, so telmisartan can be administered with or without food in these species (Boehringer Ingelheim International GmbH, 2018; FDA, 2018). However, species differences can greatly affect gastrointestinal physiology and drug absorption under different conditions. To our knowledge, no studies have evaluated the effect of feeding on the oral bioavailability of telmisartan in dogs, let alone for the TOS product. Thus, it would be prudent to determine whether a difference exists and to evaluate the magnitude of that potential effect, further enhancing rational recommendations for telmisartan administration in dogs.

The present study had two specific objectives. Our first objective was to establish the pharmacokinetic profile of telmisartan administered as TOS in healthy adult dogs at a clinically relevant dose. We hypothesized telmisartan administered as TOS would be rapidly absorbed and exhibit a moderate terminal half‐life, similar to the pharmacokinetic profile formerly established for the oral tablet formulation in dogs. Our second objective was to determine the effect of feeding on the oral absorption of telmisartan administered as TOS in dogs. We hypothesized that TOS administered to dogs with and without food would be bioequivalent based on telmisartan maximum plasma concentration (C_max_) and area under the curve (AUC).

## 2 MATERIALS AND METHODS

### 2.1 Subject recruitment

Eight healthy adult dogs weighing ≥14 kilograms were recruited in September 2021 from the pet population of the students, faculty, and staff at the University of Illinois College of Veterinary Medicine with informed owner consent. All dogs were deemed systemically healthy during an initial screening visit 2–3 weeks prior to the study. A complete physical examination was performed by a board‐certified small animal veterinary internist in addition to a complete blood count, serum biochemistry, urinalysis, and oscillometric blood pressure measurement (Cardell Insight Veterinary Monitor, Midmark Corporation). Additionally, each dog was voluntarily offered a sample of Hill's Prescription Diet® i/d canned formulation (Hill's Pet Nutrition, Inc.) and an oral bolus of water equal to the volume of TOS administered during the sampling phases of the study. Dogs were excluded if they had clinically relevant abnormal laboratory results, systolic blood pressure >160 mmHg, or were receiving any medications other than monthly heartworm and flea/tick preventatives. Dogs that were unwilling to voluntarily consume the diet or were resistant to oral liquid administration were also excluded. This study was approved by the University of Illinois Institutional Animal Care and Use Committee (protocol #19228).

### 2.2 Experimental design

This study was performed in a two‐way, balanced, cross‐over design. Dogs were randomly assigned to one of two groups by blindly drawing lots out of an envelope after successful completion of the initial screening visit. Group 1 underwent serial plasma drug monitoring following a single 1 mg/kg oral dose of telmisartan as TOS (0.1 mg/ml) on an empty stomach; then, followed by a 7‐day washout period, underwent serial plasma drug monitoring following a single oral dose of 1 mg/kg telmisartan as TOS with food. Group 2 underwent serial plasma drug monitoring following a single oral dose of 1 mg/kg telmisartan as TOS with food first, followed by administration on an empty stomach after the 7‐day washout period. The 7‐day washout period was determined based on the mean half‐life plus 3 standard deviations (5.43 h + [3 × 1.75 h] = 10.7 h) reported for dogs orally administered telmisartan tablets, which are approved for human use (Baek et al., 2013). Because the dose used in the present study was smaller and sustained‐release effects were not expected for an oral solution, we anticipated the half‐life of TOS would be similar to or shorter than what has been previously reported for the tablets. Additionally, the Food and Drug Administration recommends a washout period at least 10 times the half‐life between treatments for pharmacokinetics studies (10.7 h × 10/24 h/day = 4.5 days).

### 2.3 Drug administration and sample collection

All dogs were fasted (≥8 h) prior to arrival for catheter placement. Dogs were sedated with dexmedetomidine (Dexdomitor, Zoetis Inc.) at 4 μg/kg and butorphanol (Torbugesic‐SA, Zoetis Inc.) at 0.2 mg/kg intravenously for placement of a sterile jugular venous catheter (MILA International, Inc.) using the modified Seldinger technique (Portillo et al., 2006). Atipamezole (Antisedan, Zoetis Inc.), a reversal agent, was administered intramuscularly at 0.04 mg/kg and the dogs were allowed to recover. Following recovery, the dogs were fed a meal of a highly digestible diet (Hill's Prescription Diet® i/d) and then fasted for a minimum of 12 h overnight in hospital prior to drug administration and sampling the following morning with free access to water.

For the fasted phase, dogs were administered 1 mg/kg telmisartan as TOS. Four hours later, dogs received half of their daily energy requirement in the canned form of Hill's Prescription Diet® i/d. For the fed phase, dogs were fed as described above, followed by administration of telmisartan 5–10 min after meal completion. This protocol was adapted from the previously published TOS pharmacokinetic studies used in cats (Food and Drug Administration, 2018).

In both phases, blood samples (4 ml) were collected via the sampling catheter using the three‐syringe technique at the following time points: 0 min (before administration), 15, 30, and 45 min, 1, 1.5, 2, 4, 8, 12, 18, and 24 h. Following collection, all blood samples were placed into heparinized tubes and immediately refrigerated; plasma was separated by centrifugation at 1800 × *g* at 4°C within 4 h of collection and immediately stored at −80°C until analysis. Oscillometric blood pressure measurements were performed at the following time points: 0, 4, 8, 12, and 24 h. A systolic blood pressure <100 mmHg was considered hypotension.

### 2.4 Plasma telmisartan concentration analysis

Plasma samples were prepared by mixing 20 μl plasma with 100 μl MeOH and 10 μl 0.2 μg/ml d3‐telmisartan as the internal standard. After vortex, the mixture was subject to centrifugation for 5 min at 8000 rpm. The supernatant was for instrument injection. Samples were analyzed with the 5500 QTrap LC–MS/MS system (Sciex). Software Analyst 1.7.1 was used for data acquisition and analysis. The 1200 HPLC system (Agilent Technologies) includes a degasser, an autosampler, and a binary pump. The LC separation was performed on an Agilent SB‐Aq column (4.6 × 50 mm, 5 μm) with mobile phase A (0.1% formic acid in water) and mobile phase B (0.1% formic acid in acetonitrile). The flow rate was 0.3 ml/min. The linear gradient was as follows: 0–2 min, 100% A; 10–14 min, 0% A; 14.1–19 min, 100% A. The autosampler was set at 10°C. The injection volume was 10 μl. Mass spectra were acquired under electrospray ionization (ESI) with the ion spray voltage of +5500 V. The source temperature was 400°C. The curtain gas, ion source gas 1, and ion source gas 2 were 32, 60, and 60 psi, respectively. Multiple reaction monitoring (MRM) was used for quantitation with the following transitions: telmisartan m/z 515.1 m/z 276.1, d3‐telmisartan m/z 518.1 m/z 279.1. The linear range of the assay was 0.25–500 ng/ml. Recovery of telmisartan in blank plasma (*n* = 5) was evaluated at low (2.5 ng/ml), medium (10 ng/ml), and high (100 ng/ml) control concentrations yielding 95.9 ± 4.7%, 103.9 ± 8.3%, and 105.0 ± 7.5% recovery, respectively. Precision was also evaluated at these concentrations (*n* = 5 per concentration) on three separate days yielding intra‐ and inter‐assay coefficients of variations of 3.0–9.7% and 1.1–9.5%, respectively.

### 2.5 Pharmacokinetic and statistical analysis

Continuous data are presented as mean ± standard deviation. Non‐compartmental pharmacokinetic analysis was performed using Phoenix WinNonlin (Certara L.P.) for both the fasted and fed phases. Statistical comparisons were performed using commercially available statistical software (Prism 9, GraphPad Software Inc.). Differences in maximum concentration (C_max_), observed area under the curve (AUC_0–t_), and extrapolated area under the curve (AUC_0–∞_) between the fasted and fed phases were assessed using the 90% confidence interval bioequivalence method. Mixed effects regression models were developed for log‐transformed data of each parameter using formulation, sequence, and period as fixed effects and individual dog as a random effect. The mean squared error was obtained from the analysis of variance and used in the calculation of the 90% confidence intervals. Results are expressed as the ratio of geometric means of the fed vs. fasted phases, relative to the fasted phase, with 90% upper and lower confidence bounds. The back‐transformed difference between means is also reported. The formulations were considered bioequivalent for a parameter if the 90% confidence interval fell within 0.80–1.25 (FDA, 2006). Half‐life (*t*_1/2_), time to maximum concentration (T_max_), and mean residence time (MRT) were also compared between the fasted and fed phases and were evaluated using a Wilcoxon signed rank test as these parameters were non‐normally distributed based on the Kolmogorov–Smirnov test. *p*‐values <.05 were considered statistically significant.

The effects of phase (fasted vs. fed) and phase order on the occurrence of gastrointestinal adverse effects (vomiting, diarrhea, or both) were evaluated using a mixed effects model with phase and phase order as fixed effects and individual dog as a random effect.

## 3 RESULTS

### 3.1 Study population

Eight dogs were initially enrolled in the study. One dog in Group 1 inadvertently removed the sampling catheter shortly after time 0 sample collection in the first phase and was excluded from the study. Therefore, a total of seven adult dogs, including one intact female, four spayed females, and two neutered males were included, with a mean age of 4.4 ± 2.3 years (range 1–8 years). The mean dose of telmisartan administered throughout the study was 1.01 ± 0.004 mg/kg (range 0.99–1.02 mg/kg) (Table S1).

### 3.2 Effect of feeding on telmisartan oral solution pharmacokinetics

**FIGURE 1:**
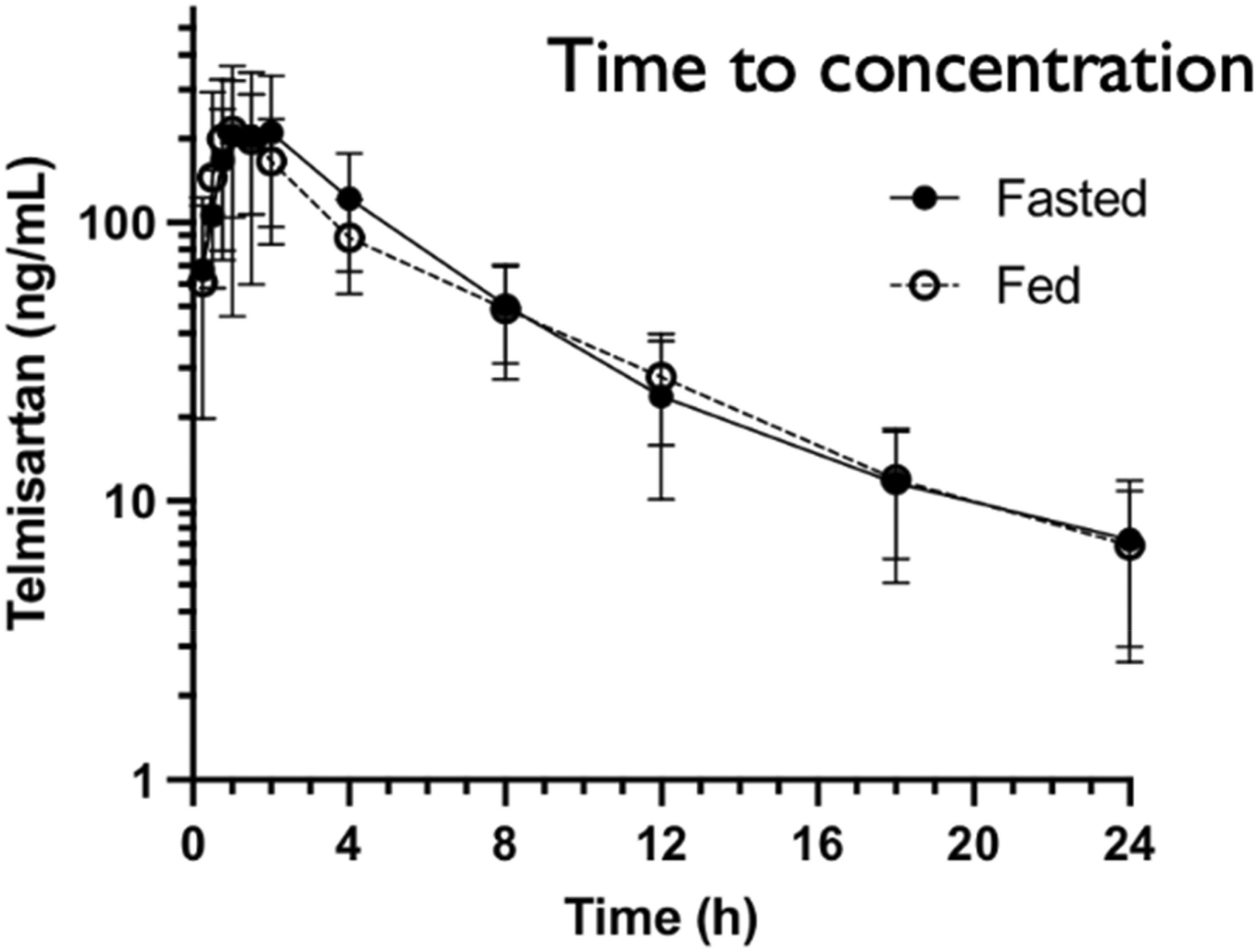
Plasma telmisartan concentration time course in dogs (*n* = 7) after oral administration of 1 mg/kg telmisartan as TOS differentiated by phase. Error bars illustrate standard deviation

Pharmacokinetic parameters for TOS with and without food are presented in Table 1. Plasma drug concentrations and pharmacokinetic parameters for individual dogs are presented in Tables S2–S5. Telmisartan concentration‐time curves were similar between fasted and fed phases (Figure 1). The ratio of the geometric means of C_max_ of the fed vs. fasted phase was 0.97 (7.48 ng/ml difference) with a 90% confidence interval of 0.74–1.27. The ratio of geometric means of the AUC_0–t_ of the fed vs. fasted phase was 0.92 (106 ng*h/ml difference) with a 90% confidence interval of 0.81–1.03. The ratio of the geometric mean of AUC_0–∞_ of the fed vs. fasted phase was 0.90 (127 ng*h/ml difference) with a 90% confidence interval of 0.82–1.00. There was no significant difference in *t*_1/2_ (*p* = .375), T_max_ (*p* > .999), or MRT (*p* = .938) between phases.

**TABLE 1:** Summary of noncompartmental analysis of pharmacokinetic parameters after single‐dose administration of TOS (1 mg/kg telmisartan) in seven dogs

	Fasted TOS				Fed TOS			
Parameter	Mean	SD	Min	Max	Mean	SD	Min	Max
C_max_ (ng/ml)	253.46	139.12	73.20	485.00	241.84	121.85	72.90	452
T_max_ (h)	1.32	0.66	0.50	2.00	1.46	1.18	0.5	4.00
MRT (h)	7.18	2.73	4.15	11.86	6.94	1.78	4.94	9.95
AUC_0–t_ (h*ng/ml)	1305.27	456.56	678.16	2110.94	1180.91	344.00	614.47	1606.57
AUC_0–∞_ (h*ng/ml)	1376.06	454.68	720.66	2169.69	1239.05	369.13	634.08	1647.72
AUC_%EXTRAP_ (%)	5.52	4.75	0.71	15.00	4.41	2.58	1.63	8.54
AUMC_0–t_ (h^2^*ng/ml)	7086.16	2397.14	4321.03	10871.73	6789.33	2455.31	4194.59	9954.55
AUMC_0–∞_ (h^2^*ng/ml)	9525.79	3683.87	4543.79	13592.81	8687.30	3717.23	4901.58	13946.44
AUMC_%EXTRAP_ (%)	22.71	13.19	4.90	46.85	19.52	8.62	10.06	29.27
*t*_1/2_ (h)	6.32	2.13	3.24	9.10	5.35	1.12	4.11	6.72
λ_z_ (1/h)	0.12	0.05	0.08	0.21	0.13	0.03	0.10	0.17

*Note*: Mean, standard deviation, minimum and maximum values are shown for each parameter of each phase (fasted/fed).

Abbreviations: AUC_%EXTRAP_, percent of AUC_0–∞_ extrapolated to infinity; AUC_0–∞_, area under the curve extrapolated to infinity; AUC_0–t_, observed area under the curve; AUMC_%EXTRAP_, percent of the AUMC_0–∞_ extrapolated to infinity; AUMC_0–∞_, area under the moment curve extrapolated to infinity; AUMC_0–t_, observed area under moment curve; C_MAX_, maximum plasma concentration; MRT, mean residence time; t_1/2_, terminal half‐life; T_MAX_, time to C_MAX_; λ_z_, terminal rate constant.

### 3.3 Adverse events

None of the seven dogs displayed clinical signs supportive of systemic hypotension, such as lethargy, weakness, confusion, or collapse or had documented evidence of hypotension following administration of the TOS product. However, a large proportion of dogs experienced mild, self‐limiting gastrointestinal upset. Neither phase (fasted vs. fed; *p* = .100) or phase order (*p* = .380) significantly impacted whether gastrointestinal signs occurred. When fasted, 3/7 dogs experienced diarrhea and 2/7 dogs developed concurrent vomiting and diarrhea. When fed, 3/7 dogs experienced diarrhea and 1/7 dog developed concurrent vomiting and diarrhea (Table S6). One of the dogs who experienced both diarrhea and vomiting when fasted was found to have ingested at least two socks prior to study admission which were evident in the vomitus. One dog also developed mild, self‐resolving generalized tremors during the fasted phase prior to receiving the TOS product. The owner reported these tremors to reflect a historical behavior in response to anxiety or need to eliminate and are not suspected to be a true adverse effect.

## 4 DISCUSSION

As telmisartan use increases in veterinary clinical practice, it is essential that rational guidelines for dosing and administration be established. Pharmacokinetic parameters have previously been published for oral telmisartan tablets (Micardis®) in dogs, albeit at higher doses (7.6–9 mg/kg) than the currently recommended starting dose (1 mg/kg q 24 h) for treating canine proteinuria (Baek et al., 2013; Brown et al., 2013). Dose directly impacts the pharmacokinetic profile of a drug; thus, it is prudent to evaluate a drug at the dosages used in clinical practice. The recently approved TOS product represents a new formulation of telmisartan that could allow for more accurate dosing in small dogs. Furthermore, an oral solution may be an attractive option for owners who struggle to administer tablets or capsules. However, formulation can impact absorption, so the previously established pharmacokinetics of the oral tablet in dogs, may not be accurate for TOS. Although TOS has already been evaluated and approved for use in cats, the pharmacokinetic parameters established in this species cannot be directly extrapolated to dogs due to the interspecies variability in drug handling (Baek et al., 2013; Food and Drug Administration, 2018). Given the critical differences in dose, product formulation, and species where telmisartan pharmacokinetics were previously investigated, there is a need to document the pharmacokinetic profile of TOS in dogs to allow better dosing recommendations in this species. Additionally, it is well known that food may interact with drug absorption through a variety of mechanisms (Welling, 1996). In humans, food reduces the bioavailability of telmisartan tablets by 6–20%, depending on dose (Boehringer Ingelheim International GmbH, 2018) and in cats, the C_max_ and AUC_0‐∞_ were higher in fasted animals administered TOS (FDA, 2018). Whether or not this is true for any telmisartan formulation in dogs is unknown, but if telmisartan absorption is greatly affected by the presence of food, it would alter recommendations for drug administration.

Our first objective was to establish the single‐dose pharmacokinetic profile of telmisartan following a 1 mg/kg oral dose administered as TOS in healthy adult dogs. We hypothesized the TOS product would exhibit a moderate terminal half‐life. In the present study, TOS exhibited a moderate mean terminal half‐life of 6.32 ± 2.13 h in the fasted state and 5.35 ± 1.12 h in the fed state. This is similar to the half‐life documented for telmisartan tablets administered to dogs (5.43 ± 1.75 h), despite being administered at a higher dose (7.6–9.0 mg/kg) (Brown et al., 2013). Additionally, another study evaluating telmisartan pharmacokinetics using a combined drug tablet (3 mg/kg telmisartan, 0.5 mg/kg amlodipine) documented a mean terminal half‐life of 5.07 h in dogs (Wang et al., 2015). Given the similar terminal half‐lives over a range of doses, it is tempting to speculate that telmisartan elimination may be a linear process in dogs at the doses evaluated. However, it is not known whether flip‐flop kinetics impacted the terminal half‐life in these studies and intravenous pharmacokinetic data for telmisartan in dogs is not publicly available for comparison. Thus, dose linearity requires further evaluation through a multiple ascending dose study design using an intravenous formulation to allow calculation of clearance. If telmisartan kinetics are linear in a clinically relevant dosing range, this would differ from that observed in humans (Israili, 2000). The terminal half‐life of telmisartan is significantly longer in people (*t*_1/2_ = >24 h) compared with dogs. Telmisartan is metabolized almost exclusively via UGT‐glucuronosyltransferases in the liver to a pharmacologically inactive metabolite (1‐*O*‐acylglucuronide) (Ebner et al., 2013; Plumb, 2019). The rate of this reaction is about 10‐fold lower in humans compared with dogs, so differences in clearance likely explain the differences in half‐life between these species (Ebner et al., 2013; Toutain & Bousquet‐Melou, 2004). This discrepancy could also be explained by extrahepatic glucuronidation in the gastrointestinal tract, alternate hepatic metabolic pathways, or a smaller volume of distribution in dogs but further studies are needed.

We also hypothesized that TOS would be rapidly absorbed in dogs, which was supported by a short T_max_ in both the fasted (1.32 ± 0.66 h) and fed state (1.46 ± 1.18 h). This is similar to the T_max_ for both the commercial telmisartan tablet (0.80 ± 0.36 h) and telmisartan‐amlodipine combined tablet (2 h) in dogs (Brown et al., 2013; Wang et al., 2015). Telmisartan T_max_ is also relatively short in humans administered tablets (0.5–1 h) and cats administered TOS (0.35 and 0.53 h, fasted vs. fed) suggesting that formulation and species do not significantly affect rate of absorption, at least for those evaluated (Food and Drug Administration, 2018; Israili, 2000). Whether or not these factors impact extent of absorption is currently unknown since absolute bioavailability has not been established in dogs for any telmisartan formulation to date.

In humans, telmisartan has high inter‐individual variability in drug concentration due to its low solubility in biological fluids, low bioavailability (30–60%), and delayed onset of action with high first‐pass metabolism following oral administration (Israili, 2000; Patel et al., 2012). High pharmacokinetic variability amongst individuals of the same species makes it challenging to recommend a dose that will be clinically efficacious for all patients. Furthermore, if high inter‐individual variability exists within the canine population, it is possible that selecting a clinically efficacious dose for all patients will be even more difficult given further possible differences in drug handling between the various breeds (Fleischer et al., 2008; Sutton, 2004). Telmisartan pharmacokinetics have not been evaluated in a large enough study of dogs to truly characterize population variability. However, we did document a threefold difference between the lowest and highest AUC and a sixfold difference in C_max_. Thus, similar inter‐individual variation in telmisartan disposition could exist in dogs with similar clinical consequences.

Our second objective was to determine the effect of feeding on the absorption of telmisartan in dogs by comparing the C_max_, AUC_0–t_, and AUC_0–∞_ between the fasted and fed state. The presence of food in the gastrointestinal tract can affect absorption based on the physiochemical properties of the specific drug by altering the pH and lipophilicity of the intestinal environment, increasing splanchnic blood flow, and prolonging gastrointestinal transit time (Welling, 1996). Species differences in gastrointestinal physiology also influence drug absorption, so administering a drug with food can significantly lower concentration in one species, while having no impact in another (Sutton, 2004). Thus, it is important to evaluate the effect of feeding on telmisartan absorption in dogs despite the available administration recommendations for humans and cats (Boehringer Ingelheim International GmbH, 2018; FDA, 2018). Using the 90% confidence interval method, the telmisartan AUC_0–t_ and AUC_0–∞_ ratios between the fasted and fed phases were within the generally accepted range of 0.80–1.25 but the C_max_ ratio (0.74–1.27) exceeded these bounds (FDA, 2006). By strict interpretive criteria, TOS administered with vs. without food cannot be considered bioequivalent. However, the C_max_ confidence interval was wide, reflecting the interindividual variability detected in our study, and fell outside both the lower and upper bounds. Therefore, we cannot make a general recommendation to administer the TOS product with or without food in dogs since the best way to maximize C_max_ is individual‐dependent. Based on AUC parameters alone, TOS administered with vs. without food is bioequivalent. As a marker for total drug exposure, AUC is sometimes considered the more important pharmacokinetic parameter, but this should be applied cautiously. Ultimately, pharmacodynamic studies are necessary to determine which parameters should be targeted (C_max_ vs. AUC vs. time within therapeutic range) to achieve the desired clinical effect. Based on these results, we suggest that TOS can be given to dogs with or without food but should be administered in the same way consistently to avoid day to day variation. Furthermore, dose should be individualized to target clinical parameters such as blood pressure or urine protein:creatinine ratio.

Although not a primary objective of our study, we did evaluate dogs administered TOS for adverse effects throughout the duration of the study period. No dogs in the present study experienced systemic hypotension which is a possible on‐target adverse effect for telmisartan.

Field studies in cats receiving 2–5 mg/kg telmisartan as TOS daily by mouth for 4–5 months documented clinical hypotension, requiring dose reductions in 24% of patients (Coleman et al., 2019; Glaus et al., 2019). The cats in these studies were geriatric and hypertensive secondary to either idiopathic hypertension, chronic kidney disease, hyperthyroidism, or multiple comorbidities. Thus, it is plausible that, the healthy, normotensive dogs utilized in our study were less susceptible to hypotension because they received a single 1 mg/kg oral dose and have assumed normal RAAS function. Interestingly, hypotension was not observed in any human field studies receiving the recommended dose (1–2 mg/kg), although it is purported as a possible adverse effect with overdose (Boehringer Ingelheim International GmbH, 2018).

Of note, all dogs in our study experienced mild, transient gastrointestinal upset throughout one or both sampling phases. No notable differences were observed between phases, order of phases or individual study participants. Mild, large bowel diarrhea was the predominant adverse effect, impacting all seven dogs. Three individual dogs vomited once and only two dogs experienced gastrointestinal upset in both phases. It is unclear if the high incidence of diarrhea and vomiting represent a true adverse reaction because the adverse effect profile for TOS in dogs is unknown due to the paucity of available literature (Plumb, 2019). A major consideration for the gastrointestinal signs observed in our study may be related to the abrupt dietary change without an acclimation period. An acclimation period was not considered necessary initially due to selecting an easily digestible diet formulated for dogs with pre‐existing gastrointestinal disturbances. However, we cannot exclude this possibility until this study has been revised, with an appropriate acclimation period to assess for repeatability. Consideration is also given to effects of sedative drugs, TOS formulation (viscous solution), historical dietary indiscretion, environmental stress, and/or a combination of the multiple factors listed above. In field trials of TOS in cats, the most common adverse effects were weight loss (34.6%), vomiting (29.9%), dehydration (16.8%), and diarrhea (11.2%), so it is quite plausible that vomiting and diarrhea are true adverse effects of TOS in dogs, rather than a result of our study design (Food and Drug Administration, 2018).

Our study has several limitations that should be considered. As with many veterinary pharmacokinetics studies, our sample size was small, which may have increased our risk of a type II statistical error. Using a two‐sample equivalence power calculator (powerandsamplesize.com), the power to detect bioequivalence between the fasted and fed phases for C_max_, AUC_0‐t_, and AUC_0–∞_ ranged from 0.75 to 0.76. Another important consideration is that we recruited healthy client‐owned dogs in this study rather than using purpose‐bred animals. Although pharmacokinetic studies enrolling dogs of varying age, sex and breed have the potential to introduce significant variability in the data generated, this group of dogs may better represent the canine population in whom TOS will have the greatest clinical use compared with using purpose‐bred dogs. Furthermore, we selected healthy dogs to better characterize the pharmacokinetic profile of the recommended starting dose of telmisartan (1 mg/kg q 24 h) to provide the initial framework for translating this data to dogs with clinical illness. Finally, we did not perform follow‐up biochemical analyses, with specific regard to their renal and electrolyte parameters, which is a documented adverse effect of RAAS inhibition common in dogs and rare in cats and humans (Brown et al., 2013; Food and Drug Administration, 2018; Sadjadi et al., 2009). However, these values were not expected to be abnormal given the one‐time administration to healthy adult dogs (Schierok et al., 2001).

Additional studies evaluating TOS and other telmisartan products in dogs are warranted. The present study did not evaluate the effect of various dietary compositions on absorption, specifically evaluating canned versus kibble formulations or their macronutrient profile, which may play a more important role in bioavailability. Larger pharmacokinetic studies using population‐based approaches are needed to assess variability in healthy dogs and, particularly, in clinically ill patients with various systemic diseases. Multidose pharmacokinetic studies would also provide more accurate information about plasma drug concentrations at steady state. The current recommended dose of telmisartan in dogs (1 mg/kg q 24 h) is based on expert opinion (Brown et al., 2013). Thus, pharmacodynamic studies evaluating markers of RAAS inhibition are ultimately needed to determine rational dosing recommendations in dogs, with follow‐up clinical studies evaluating the overall success of treatment and documented incidence of adverse effects. Our study provides the initial framework to advance our understanding of TOS in dogs as it becomes more popular in clinical practice.

## 5 CONCLUSION

The results of our study demonstrate that single dose pharmacokinetic parameters of TOS appear similar to those for telmisartan tablets in dogs. Thus, TOS can probably be used in a similar manner in clinical animals with the benefit of allowing for more accurate dosing in small dogs and providing a liquid option for owners who struggle to administer tablets or capsules. Although not bioequivalent based on C_max_, TOS can likely be administered with or without food in dogs as long the drug is administered the same way each day to promote consistent absorption. Additional pharmacokinetic and pharmacodynamic studies are warranted to further develop rational recommendations for telmisartan use in canine medicine.

## AUTHOR CONTRIBUTIONS

JR and ZL conceived this study. All authors contributed to its execution and data analysis. AB wrote the initial manuscript draft and all authors contributed to and approved the final version.

## FUNDING INFORMATION

Funding was provided through the Department of Veterinary Clinical Medicine at the University of Illinois College of Veterinary Medicine in Urbana, Illinois.

## CONFLICT OF INTEREST

We have no financial conflicts of interest or disclaimers to disclose.

## ETHICAL APPROVAL

The authors confirm that the ethical policies of the journal, as noted on the journal's author guidelines page, have been adhered to and the appropriate ethical review committee approval has been received. The authors confirm that they have adhered to either US or European standards for the protection of animals used for scientific purposes.

## Supporting information


Table S1–S6

## Data Availability

The data that supports the findings of this study are available in the supplementary materials of this article.
