# Supplementary material for: Single‐dose pharmacokinetics of telmisartan oral solution and effect of feeding in dogs
Source: J Vet Pharmacol Ther. 2022 Nov 10;46(1):17–24. doi: 10.1111/jvp.13104 (PMC10098839; doi:10.1111/jvp.13104)
Supplement: Supplementary file 1 — Table S1–S6 [file JVP-46-17-s001.docx]

**Table S1** – Demographic data for individual dogs participating in study. The phase order highlights the randomized order conducted for each dog. The weight reported is the dog’s weight on the day of telmisartan oral solution (TOS) administration for both phases.

| **Study ID** | **Phase Order** | **Age (y)** | **Sex** | **Breed** | **Weight (kg)** |
| --- | --- | --- | --- | --- | --- |
| **1.1** | Fast/Fed | 8 | FI | Catahoula | 21.8, 21.9 |
| **1.3** | Fed/Fast | 4 | FS | Standard Poodle | 16.8, 16.2 |
| **1.4** | Fed/Fast | 6 | FS | Labrador Retriever | 23.0, 22.6 |
| **2.1** | Fast/Fed | 1 | MN | Siberian Husky | 27.5, 28.0 |
| **2.2** | Fast/Fed | 5 | FS | Labrador Retriever | 25.1, 25.3 |
| **2.3** | Fed/Fast | 2 | FS | Rhodesian Ridgeback | 33.0, 32.7 |
| **2.4** | Fed/Fast | 5 | MN | Mixed breed dog | 23.7, 23.5 |

**Note:** y = years; kg = kilograms; FI = female intact; FS = female spayed; MN = male neutered

**Table S2** – Plasma telmisartan oral solution (TOS) concentrations for individual dogs following a single oral dose (1 mg/kg) after fasting for 12 hours.

| **Time (h)**  **Dog** | **Plasma TOS Concentration (ng/mL)** | | | | | | |
| --- | --- | --- | --- | --- | --- | --- | --- |
|  | **1.1** | **1.3** | **1.4** | **2.1** | **2.2** | **2.3** | **2.4** |
| **0** | 0 | 0 | 0 | 0 | 0 | 0 | 0 |
| **0.25** | 26.4 | 145 | 24.7 | 98.7 | 41 | 104 | 33.1 |
| **0.5** | 55.2 | 180 | 85.5 | 107 | 52 | 157 | 108 |
| **0.75** | 138 | 156 | 138 | 104 | 73.2 | 336 | 226 |
| **1** | 150 | 153 | 103 | 106 | 66.8 | 377 | 485 |
| **1.5** | 179 | 124 | 133 | 136 | 66 | 303 | 483 |
| **2** | 279 | 71.1 | 172 | 208 | 48.4 | 285 | 407 |
| **4** | 113 | 58.2 | 126 | 206 | 48.8 | 145 | 158 |
| **8** | 30.1 | 44.7 | 84.8 | 53.3 | 53.2 | 28.1 | 58.7 |
| **12** | 11.3 | 26.6 | 32.3 | 12.5 | 18.8 | 15.6 | 49.5 |
| **18** | 2.61 | 20 | 17.1 | 7.37 | 8.4 | 8.27 | 17.5 |
| **24** | 1.66 | 13.2 | 13.4 | 5.01 | 4.82 | 4.06 | 8.58 |

**Table S3** – Plasma telmisartan oral solution (TOS) concentrations for individual dogs following a single oral dose (1 mg/kg) after feeding half their calculated resting energy requirement (RER).

| **Time (h)**  **Dog** | **Plasma TOS Concentration (ng/mL)** | | | | | | |
| --- | --- | --- | --- | --- | --- | --- | --- |
|  | **1.1** | **1.3** | **1.4** | **2.1** | **2.2** | **2.3** | **2.4** |
| **0** | 0 | 0 | 0 | 0 | 0 | 0 | 0 |
| **0.25** | 31.4 | 195 | 42.8 | 77.9 | 17.8 | 33 | 28.7 |
| **0.5** | 48 | 452 | 85.2 | 229 | 49.1 | 88.1 | 67.9 |
| **0.75** | 56.1 | 442 | 106 | 275 | 163 | 170 | 188 |
| **1** | 72.9 | 391 | 99.2 | 271 | 199 | 191 | 275 |
| **1.5** | 60.8 | 299 | 80 | 232 | 231 | 262 | 224 |
| **2** | 51.4 | 215 | 86.3 | 198 | 177 | 232 | 202 |
| **4** | 40.3 | 71.2 | 125 | 130 | 64.5 | 85.9 | 101 |
| **8** | 48.7 | 36.1 | 66.6 | 83 | 28.3 | 23 | 56.9 |
| **12** | 19.3 | 26.3 | 47 | 35 | 14.3 | 17 | 36 |
| **18** | 6.08 | 11.3 | 20.7 | 13.2 | 5.96 | 8.36 | 18.1 |
| **24** | 3.31 | 8.01 | 12.8 | 6.59 | 3.96 | 2.69 | 11.1 |

**Table S4** – Noncompartmental analysis of individual pharmacokinetic parameters after single-dose administration of telmisartan oral solution (TOS) (1 mg/kg) after fasting for 12 hours.

| **Parameter** | **Dog** | | | | | | |
| --- | --- | --- | --- | --- | --- | --- | --- |
|  | **1.1** | **1.3** | **1.4** | **2.1** | **2.2** | **2.3** | **2.4** |
| **C_MAX_ (ng/mL)** | 279 | 180 | 172 | 208 | 73.2 | 377 | 485 |
| **T_MAX_ (h)** | 2 | 0.5 | 2 | 2 | 0.75 | 1 | 1 |
| **MRT (h)** | 4.15 | 11.86 | 8.72 | 6.30 | 8.72 | 4.68 | 5.85 |
| **AUC_0-t_ (h*ng/mL)** | 1085.94 | 974.5 | 1403.68 | 1398.13 | 678.16 | 1485.58 | 2110.94 |
| **AUC_0-∞_ (h*ng/mL)** | 1093.71 | 1146.49 | 1516.23 | 1463.88 | 720.66 | 1521.77 | 2169.69 |
| **AUC_%EXTRAP_ (%)** | 0.71 | 15.00 | 7.42 | 4.49 | 5.90 | 2.38 | 2.71 |
| **AUMC_0-t_ (h^2^*ng/mL)** | 4321.03 | 7224.13 | 9581.68 | 6785.07 | 4888.58 | 5930.93 | 12683.97 |
| **AUMC_0-∞_ (h^2^*ng/mL)** | 4543.79 | 13592.81 | 13228.34 | 9226.25 | 6283.13 | 7122.24 | 12683.97 |
| **AUMC_%EXTRAP_ (%)** | 4.90 | 46.85 | 27.57 | 26.46 | 22.20 | 16.73 | 14.29 |
| **t_1/2_ (h)** | 3.24 | 9.03 | 5.82 | 9.10 | 6.11 | 6.18 | 4.75 |
| **λ_z_ (1/h)** | 0.21 | 0.08 | 0.12 | 0.08 | 0.11 | 0.11 | 0.15 |

**Note:** C_MAX_, maximum plasma concentration; T_MAX_, time to C_MAX_; MRT, mean residence time; AUC_0-t_, observed area under the curve; AUC_0-∞_, area under the curve extrapolated to infinity; AUC_%EXTRAP_, percent of AUC_0-∞_ extrapolated to infinity; AUMC_0-t_, observed area under moment curve; AUMC_0-∞_, area under the moment curve extrapolated to infinity; AUMC_%EXTRAP_, percent of the AUMC_0-∞_ extrapolated to infinity; t_1/2_, terminal half-life; λ_z_, terminal rate constant.

**Table S5** – Noncompartmental analysis of individual pharmacokinetic parameters after single-dose administration of telmisartan oral solution (TOS) (1 mg/kg) after feeding half their calculated resting energy requirement (RER).

| **Parameter** | **Dog** | | | | | | |
| --- | --- | --- | --- | --- | --- | --- | --- |
|  | **1.1** | **1.3** | **1.4** | **2.1** | **2.2** | **2.3** | **2.4** |
| **C_max_ (ng/mL)** | 72.9 | 452 | 125 | 275 | 231 | 262 | 275 |
| **T_max_ (h)** | 1 | 0.5 | 4 | 0.75 | 1.5 | 1.5 | 1 |
| **MRT (h)** | 7.73 | 5.94 | 9.95 | 6.26 | 5.50 | 4.94 | 8.27 |
| **AUC_0-t_  (h*ng/mL)** | 614.47 | 1418.46 | 1282.58 | 1606.57 | 894.69 | 1058.33 | 1391.28 |
| **AUC_0-∞_  (h*ng/mL)** | 634.08 | 1491.55 | 1402.27 | 1647.72 | 922.91 | 1075.84 | 1498.65 |
| **AUC_%EXTRAP_ (%)** | 3.09 | 4.90 | 8.54 | 2.50 | 3.06 | 1.63 | 7.18 |
| **AUMC_0-t_  (h^2^*ng/mL)** | 4314.88 | 6444.66 | 9954.55 | 9071.76 | 4194.59 | 4776.44 | 8768.46 |
| **AUMC_0-∞_  (h^2^*ng/mL)** | 4901.58 | 8865.79 | 13946.44 | 10316.43 | 5072.90 | 5310.60 | 12397.37 |
| **AUMC_%EXTRAP_ (%)** | 11.97 | 27.31 | 28.62 | 12.06 | 17.31 | 10.06 | 29.27 |
| **t_1/2_ (h)** | 4.11 | 6.32 | 6.48 | 4.33 | 4.94 | 4.51 | 6.72 |
| **λ_z_ (1/h)** | 0.17 | 0.11 | 0.11 | 0.16 | 0.14 | 0.15 | 0.10 |

**Note:** C_MAX_, maximum plasma concentration; T_MAX_, time to C_MAX_; MRT, mean residence time; AUC_0-t_, observed area under the curve; AUC_0-∞_, area under the curve extrapolated to infinity; AUC_%EXTRAP_, percent of AUC_0-∞_ extrapolated to infinity; AUMC_0-t_, observed area under moment curve; AUMC_0-∞_, area under the moment curve extrapolated to infinity; AUMC_%EXTRAP_, percent of the AUMC_0-∞_ extrapolated to infinity; t_1/2_, terminal half-life; λ_z_, terminal rate constant.

**Table S6** – Number of dogs having gastrointestinal adverse events separated by phase, period, and type of adverse effect.

| **PHASE** | **PERIOD 1** | | **PERIOD 2** | | **Total** |
| --- | --- | --- | --- | --- | --- |
|  | **Diarrhea** | **Vomiting & Diarrhea** | **Diarrhea** | **Vomiting & Diarrhea** |  |
| **Fasted** | 1/7 | 1/7 | 2/7 | 1/7 | 5/7 |
| **Fed** | 2/7 | 0/7 | 1/7 | 1/7 | 4/7 |
| **Total** | 3/7 | 1/7 | 3/7 | 2/7 | 9/14 |

**Note:** Phase = fasted or fed; Period = first vs. second TOS administration and sampling time period (separated by 7-day washout period)
